# Scarred for Life as Covid-19 Leaves Its Mark: An Autobiographical Case Report

**DOI:** 10.7759/cureus.21542

**Published:** 2022-01-24

**Authors:** Monjuri Borkotokey

**Affiliations:** 1 Biochemistry, Lokopriya Gopinath Bordoloi (LGB) Regional Institute of Mental Health, Tezpur, IND

**Keywords:** long covid, auto-immune disease, arrhythmia, covid-19, autobiographical case report

## Abstract

The COVID-19 pandemic affected humans in many more ways than one. The medical fraternity worked relentlessly, clad in personal protective equipment (PPE), to fight a virus that had taken the world by surprise. The irony is that, despite the PPE, our fraternity never felt so vulnerable and exposed. Yet, they stood out in handling the COVID-19 pandemic with due diligence. This case report describes the wearied experience of a healthcare worker affected by COVID-19 and its effect on physical and mental health. The author describes her experience as she suffered from COVID-19 and long COVID.

## Introduction

Doctors and nurses worldwide have worked tirelessly over the past two years, many times putting their families at risk of spreading the disease despite all efforts. Yet, they stood out in handling the COVID-19 pandemic with due diligence, despite the limitations in resources and manpower, especially in India, where the population is overwhelming. The mental health of healthcare workers has been severely affected in this period because of the increased workload, heightened media reports, uncertainty about the newer medicines, and frequently changing guidelines on treatment protocols [1,2]. It has been seen that a substantial proportion of survivors of COVID-19 suffer from long-term or post-COVID symptoms, which substantially affect their physical and mental health, impacting their professional lives [3]. Here is my COVID-19 experience, both during the hospitalization and the period thereafter.

## Case presentation

**Figure 1:**
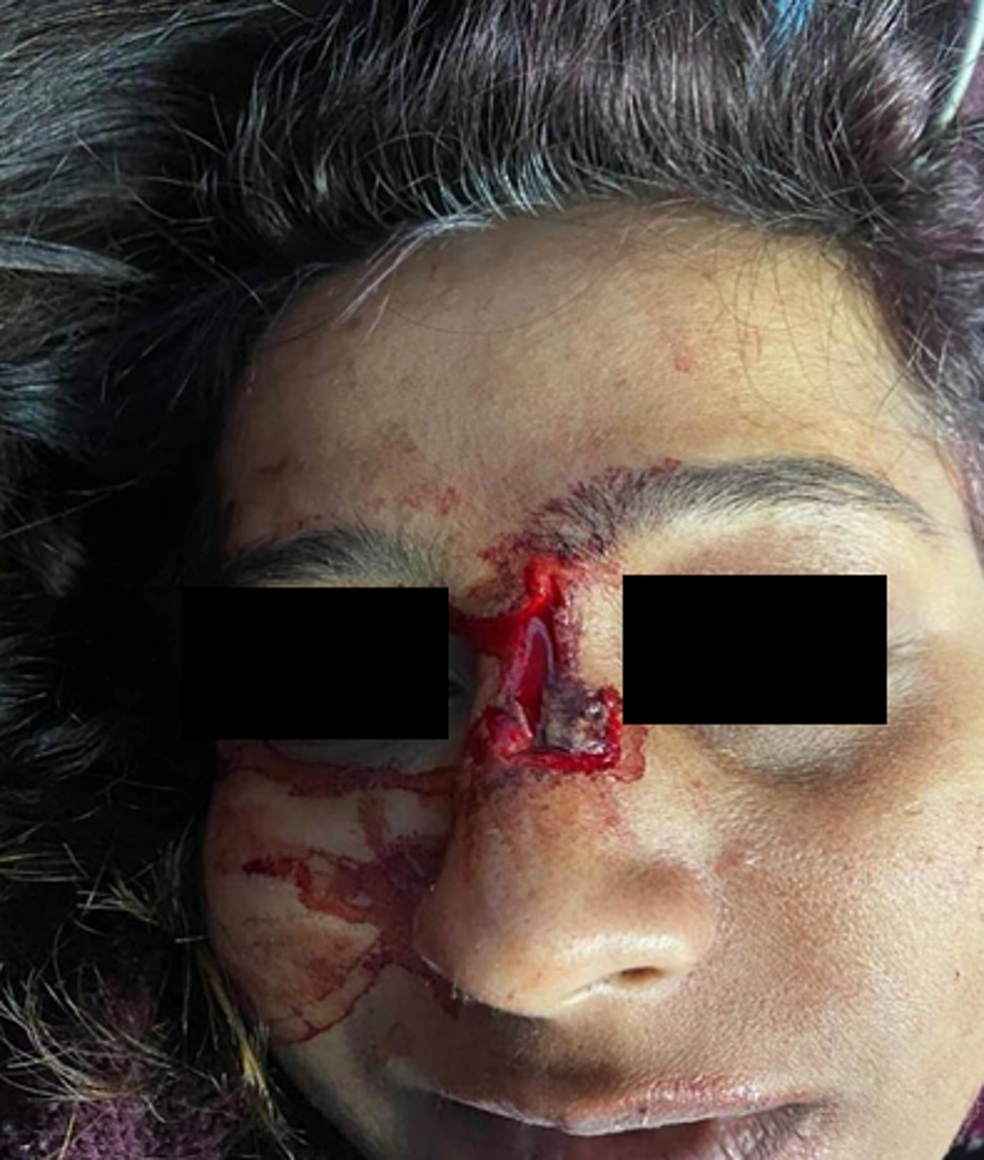
The lacerated cut injury over the bridge of nose

It all started in June 2020, when my husband was made in charge of the COVID ICU at a tertiary care medical institute in North East India. This was the time when the first wave of COVID-19 was at its peak in India. Hospitals were fully occupied, and the anesthesiologists and intensivists were at the helm of affairs. The responsibility of covering a COVID ICU round the clock was taking a toll on his health. One fine morning, my husband complained of body aches and a fever. What followed next was exasperating; having to wait patiently for the RT-PCR test report, isolating at home, and the most difficult part was making my four-year-old son stay away from his father. To make matters worse, my son developed a fever and loose stools, and refused to eat or drink anything. In the next 24 hours, we were all admitted to the COVID ward. My husband and son’s RT-PCR reports were positive, while mine was negative. I developed breathing difficulty and palpitations in the next couple of days. I would like to apprise the readers here that I have a history of auto-immune uveitis, having had several relapses for which I was on steroids and Azathioprine in the past. The physician treating me was aware of my past history and started me on steroids and low-molecular-weight heparin (LMWH). The tests for inflammatory markers like C-reactive protein (CRP), D-dimer, and ferritin were all ordered, and so were chest X-ray, ECG, echocardiography, and CK-MB as I continued to have dyspnea and palpitations. Throughout this, the oxygen saturation (SpO_2_) was normal, maintained above 94% in room air. Over the next 48 hours, I began to feel better as I regained my strength and the dyspnea settled down. The steroids were stopped by the fifth day. The next day, I had a fever. It worsened by the evening, and I began to have a sinking feeling, completely unaware of my surroundings. Tests were re-run to rule out secondary bacterial infections; high-resolution computed tomography (HRCT) thorax was done to rule out pulmonary lesions. The CRP was elevated, the neutrophil-lymphocyte (NL) ratio was above 4, and the infective panel was normal. After a detailed discussion of the physician with my husband, the symptoms were attributed to the sudden withdrawal of steroids. The sudden flare of symptoms was due to the inflammatory hyperresponsiveness due to an underlying autoimmune disorder. With re-initiation of dexamethasone, my symptoms improved dramatically; by the 14th day, I tested negative and was discharged home. Over the next few days, we were back to our routine; both I and my husband joined our work. Initially, it was just weakness and easy fatigability, which gradually transitioned into palpitations and having a sensation of a "missed" heartbeat, especially at night. I attributed my symptoms to the delayed effects of COVID-19 and believed that they would settle down on their own. This went on for almost three months, with more frequent episodes of palpitations when I decided to go for a checkup. The cardiologist ordered an ECG, echocardiography, and Holter Monitoring. The latter revealed around 848 premature ventricular contractions (PVC) over a 24-hour study period. I was told to observe my symptoms, take magnesium supplements, and review them after a month if symptoms do not abate. I was once again assured that these arrhythmias were benign and that they would settle down on their own. I wish that they were true; almost a month later, late at night while I was in the washroom, I had an uneasy feeling and hurriedly opened the bathroom door and called out for my husband. Before I could realize what had happened, I was lying in a pool of blood, having collapsed on the floor after hitting my head against the edge of my bed. I was immediately taken to the emergency department, where initial resuscitation was done, and later I was shifted to the Cardiology Critical Care Unit (CCU). I had sustained a deep lacerated cut injury over the bridge of my nose with an underlying fracture of the nasal bone (Figure 1).

**Figure 2:**
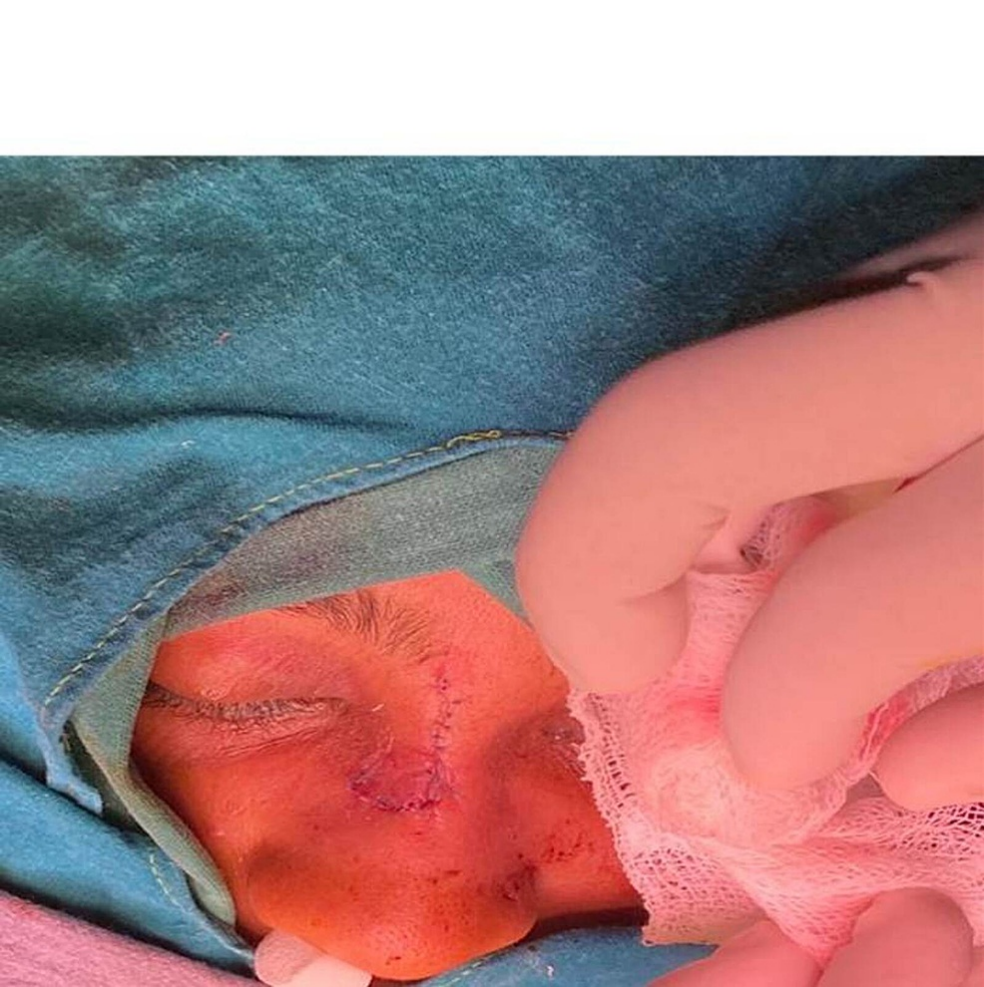
The suture site in the immediate post operative period

The bleeding had stopped and I managed to remain calm while I could sense the worry on my husband’s face. The CT scan of the brain was normal, and I was monitored in the CCU; while I was hemodynamically stable, the arrhythmias kept coming periodically. I was operated upon by a plastic surgeon with assistance from an ENT surgeon and was anesthetized by my husband. The perioperative course was uneventful, and I waited eagerly for the bandages to be removed. I had a scar right across the bridge of my nose, and despite the assurances from the surgical team that the scar would fade eventually, I realized that I would have to get used to living with a scar (Figure 2). I was started on bisoprolol and diligently monitored my blood pressure over the ensuing months. I went to a referral hospital for an electrophysiology study of the heart, where aberrant conduction abnormalities were ruled out and the episode was attributed to being neurocardiogenic syncope. It has been close to nine months since the incident; I am currently on metoprolol and monitor my symptoms regularly.

## Discussion

The manifestations of COVID-19 range from asymptomatic infection to flu-like symptoms in the majority of the population. In the susceptible population, disease severity could result in respiratory tract involvement and multi-organ dysfunction requiring mechanical ventilation and organ support.

It has been observed that immune-mediated mechanisms could lead to the complications of COVID-19. The hyperinflammatory response seen in individuals affected by COVID-19 is similar to the pathogenesis of the autoimmune disease. Autoantibodies have been identified in patients with symptomatic COVID-19 infections [4]. A possible mechanism for disease manifestations and an immune response is a structural similarity between the host cells and the virus [5]. The inflammatory markers like IL-6 and C-reactive protein (CRP) indicate the ongoing inflammation [6], which were elevated initially in my case and gradually subsided with steroid therapy. My past history of auto-immune uveitis could have led to more disease severity in my case.

COVID-19 affects the cardiovascular system causing myocardial injury, through interaction via the angiotensin-converting enzyme 2 (ACE-2) receptor [7]. Endothelial damage-causing endocarditis and myocarditis have been noted in individuals in whom the respiratory system is relatively spared. There have been numerous reports of new-onset cardiovascular symptoms in patients without any prior history of cardiac ailments [8]. The probable mechanism for myocardial damage can be due to the massive release of cytokines, causing a cytokine storm, resulting in injury to the vascular endothelium and myocardial cells [9,10]. As previously mentioned, SARS-CoV-2 interacts with ACE2 receptors located in the heart and vascular endothelium, causing injury to the myocardium and leading to myocarditis [11]. It has been observed that patients infected by SARS-CoV-2 have arrhythmias, especially in individuals with hypertension, diabetes mellitus, or previous cardiac disease. Acute derangement of electrolytes like hypokalemia can predispose to various types of cardiac rhythm disturbances [9]. Various factors that can contribute to arrhythmias include low oxygen saturation, hypotension predisposing to impaired coronary perfusion, inflammatory response, and effects of certain medications like azithromycin that were initially used to manage COVID-19 patients [12].

Long COVID-19 has been defined as the presence of signs and symptoms that cannot be attributed to other causes, four weeks after being diagnosed with SARS-Cov-2 infection [13]. The clinical features may fluctuate and can affect any organ system and may persist for months. In a global survey by Gopinathannair et al. [14], numerous arrhythmic events were noted in hospitalized COVID-19 patients. They reported that the most common supraventricular and ventricular arrhythmias were atrial fibrillation and monomorphic premature ventricular contractions, respectively. It is not clear how long the arrhythmias will remain.

The occurrence of syncope is usually attributed to orthostatic hypotension. The usual mechanism of syncope is primarily due to an imbalance between the parasympathetic and sympathetic nervous systems, with vagal predominance. It has been reported that sinus tachycardia, which in my case was persistent post-COVID, can contribute to arrhythmias and syncope [15].

## Conclusions

The medical fraternity is at the forefront of the fight against the virus. Every morning, the scar reminds me that I fought this battle valiantly and my family stood by me through the difficult times. COVID-19 has taught humanity that, among many things, nothing is more precious than family. The long-term consequences of post-COVID symptoms may continue to affect our physical, mental, and societal well-being for a very long time. While vaccination has helped slow down the pandemic, further research is warranted for conditions like long-term COVID, which will continue to baffle clinicians and researchers alike.
